# Editorial: Dynamics of Sensorimotor Interactions in Embodied Cognition

**DOI:** 10.3389/fpsyg.2015.01929

**Published:** 2016-01-05

**Authors:** Guillaume T. Vallet, Lionel Brunel, Benoit Riou, Nicolas Vermeulen

**Affiliations:** ^1^Department of Psychology, Centre de Recherche de l'Institut Universitaire de Gériatrie de MontréalMontreal, Canada; ^2^Laboratoire EPSYLON, Department of Psychology, Université Paul-Valéry Montpellier IIIMontpellier, France; ^3^Laboratoire d'Etude des Mécanismes Cognitifs, Department of Psychology, Université Lyon 2Lyon, France; ^4^Psychological Sciences Research Institute, Université Catholique de LouvainLouvain-la-Neuve, Belgium

**Keywords:** embodied cognition, grounded cognition, situated cognition, sensorimotor interactions, memory, action, perception, emotion

The concept of incorporating the current situation and the body state within cognitive processes, referred to as embodiment, has revolutionized cognitive research (Glenberg et al., 2013). Interest in this approach has grown substantially in the last few decades. Embodied cognition has now been demonstrated across a wide range of topics, from babies (e.g., Smith and Gasser, 2005) to elderly adults (e.g., Vallet et al., 2013a), from normal cognition to neuropsychology (e.g., Vallet et al., 2013b) as well as in emotion (e.g., Vermeulen et al., 2007), and in neuroscience as a whole (e.g., Pulvermüller, 2013). Nevertheless, there is yet much to discover in order to better understand embodiment.

One of the striking arguments of embodiment is that sensori-motor features are at the core of mental processes (Pecher and Zwaan, 2005; Barsalou, 2010). Their relationship should also be considered to be dynamic. In the embodied cognition approaches, perception, memory, and action are no longer regarded as (relatively) independent functions, but rather as closely interacting components. Some authors even argue for an overlap within these processes (Brunel et al., 2009, 2015; Vermeulen et al., 2009) that would rely on the same neural code (e.g., Hommel). One key direction in embodiment that needs to be further explored is the dynamic interaction between the sensory and motor components across the different cognitive functions. The present Research Topic aims to shed new light on this issue.

The importance of studying this problematic is well presented in Dijkstra and Post's critical review. Their article highlights the crucial role of sensorimotor simulation across several cognitive activities (e.g., reasoning, evaluating) and how the interaction between the current situation and the simulation of past sensorimotor states mediates the emergence of adaptive behavior. This is possible since perception and memory interact closely and share processes and resources (e.g., Vermeulen et al., 2008; Riou et al., 2011; Rey et al., 2014). In other words, “direct” perception of an object and the mental simulation of this object involve common sensorimotor units. Heurley and Ferrier argue in their perspective that these interactions serve to plan and control actions to interact with well-known objects in non-optimal perceptual conditions, thus producing adaptive behavior.

The idea that cognitive activities are strongly influenced by the given social and physical environment is referred to as situated cognition. Rey et al. demonstrate that motor simulation relies on the relevance of the object as a function of the task. Further evidence also supports the hypothesis that sensorimotor simulation is heavily influenced by situational aspects (Kapoula et al.). For instance, Amorim et al. have demonstrated that the visual angle and screen orientation along with contextual information modulates the resulting movement.

The reverse is also applicable, since sensorimotor simulation shapes our perception of, as well as our interaction with, our environment. Grade et al. found that similar action simulation underlies both reachability and egocentric distance perception. This effect can be generalized across individuals and possibly incorporate the role of social environment in cognition. In accordance, Quesque and Coello show that during reach-to-grasp actions, participants unconsciously modify their trajectory curvature based on their partner's eye level. This adaptation to biological motion is one the key component of social interaction, but human movements are so complex that they might be described as mathematically chaotic. Nonetheless, children quickly develop the ability to coordinate gaze, and in some respect posture, in response to complex chaotic motion structures (Haworth et al.).

The dynamics of sensorimotor interactions allows for situational behavioral adaptation in reaching and grasping, but also in more complex evaluative processes. For instance, associations between handedness and valence have previously been found (e.g., Casasanto, 2011). de la Vega et al. extend this finding by showing that strong right-footers respond to positive words faster with the dominant foot. The association between valence and laterality, however, becomes less clear when motor fluency is taken into account. Brouillet et al. observe that most participants prefer choosing stable supports for “good” items, regardless of side and handedness. The space-valence association could also be reported for the vertical axis. Xie et al. demonstrate that the processing of affective valence concepts activated the vertical spatial axis (positive in the up position).

The association between cognition and space in the context of emotion is well documented, but it is not restricted to this cognitive domain. Hartmann et al. report that simple arithmetics are associated with gaze shifting along the vertical axis. Interestingly, operand magnitude partly modulated horizontal gaze position as well. The reciprocal influence of motor components on numerical cognition may also provide the opportunity to assess numerical cognition with methods such as mouse tracking. In their perspective, Fischer and Hartmann point out important insight and methodological considerations on conceptual aspects related to numeric cognition. This perspective has been further elaborated by Faulkenberry and Rey.

These recent reports offer a new perspective on cognitive functioning, one that combines sensorimotor dynamics with contextual and body information. These new concepts provide new opportunities to explore related domains such as motivation (Shalev) and anticipation (Raab) and offer a new framework to interpret well-known and sometimes contradictory results in fields such as short-term memory (Macken et al.) or healthy cognitive aging (Vallet).

## Author contributions

Every author participates to the writing of the editorial article. All of them also significantly contribute to the edition of the articles of the research topic.

## Funding

GV is supported by a postdoctoral grant from the Fonds Québécois de la Recherche en Santé (FRQS). LB is supported by ACCEPT (“Assistance Tools and Cognitive Contribution: Embodied Potential of Technology”), French research ministerial mission (MiRe-DREES and CNSA). BR is supported by FS-CH (Formation Universitaire à distance, Suisse) and this work by the LabEx Cortex (“Construction, Function, and Cognitive Function and Rehabilitation of the Cortex,” ANR-10-LABX-0042) of Université de Lyon, within the program “Investissements d'Avenir” (ANR-11-IDEX-0007) operated by the French National Research Agency (ANR).

### Conflict of interest statement

The authors declare that the research was conducted in the absence of any commercial or financial relationships that could be construed as a potential conflict of interest.
